# Internet-Delivered Interpersonal Psychotherapy Versus Internet-Delivered Cognitive Behavioral Therapy for Adults With Depressive Symptoms: Randomized Controlled Noninferiority Trial

**DOI:** 10.2196/jmir.2307

**Published:** 2013-05-13

**Authors:** Tara Donker, Kylie Bennett, Anthony Bennett, Andrew Mackinnon, Annemieke van Straten, Pim Cuijpers, Helen Christensen, Kathleen M Griffiths

**Affiliations:** ^1^Black Dog InstituteUniversity of New South WalesSydneyAustralia; ^2^Centre for Mental Health ResearchThe Australian National UniversityCanberraAustralia; ^3^Orygen Youth HealthUniversity of MelbourneMelbourneAustralia; ^4^Clinical PsychologyDepartment of Psychology and EducationVU University of AmsterdamAmsterdamNetherlands; ^5^EMGO Institute for Health and Care ResearchVU University and VU University Medical Center AmsterdamAmsterdamNetherlands

**Keywords:** interpersonal relations, cognitive behavior therapy, depressive disorder, Internet, randomized controlled trial

## Abstract

**Background:**

Face-to-face cognitive behavioral therapy (CBT) and interpersonal psychotherapy (IPT) are both effective treatments for depressive disorders, but access is limited. Online CBT interventions have demonstrated efficacy in decreasing depressive symptoms and can facilitate the dissemination of therapies among the public. However, the efficacy of Internet-delivered IPT is as yet unknown.

**Objective:**

This study examines whether IPT is effective, noninferior to, and as feasible as CBT when delivered online to spontaneous visitors of an online therapy website.

**Methods:**

An automated, 3-arm, fully self-guided, online noninferiority trial compared 2 new treatments (IPT: n=620; CBT: n=610) to an active control treatment (MoodGYM: n=613) over a 4-week period in the general population. Outcomes were assessed using online self-report questionnaires, the Center for Epidemiological Studies Depression scale (CES-D) and the Client Satisfaction Questionnaire (CSQ-8) completed immediately following treatment (posttest) and at 6-month follow-up.

**Results:**

Completers analyses showed a significant reduction in depressive symptoms at posttest and follow-up for both CBT and IPT, and were noninferior to MoodGYM. Within-group effect sizes were medium to large for all groups. There were no differences in clinical significant change between the programs. Reliable change was shown at posttest and follow-up for all programs, with consistently higher rates for CBT. Participants allocated to IPT showed significantly lower treatment satisfaction compared to CBT and MoodGYM. There was a dropout rate of 1294/1843 (70%) at posttest, highest for MoodGYM. Intention-to-treat analyses confirmed these findings.

**Conclusions:**

Despite a high dropout rate and lower satisfaction scores, this study suggests that Internet-delivered self-guided IPT is effective in reducing depressive symptoms, and may be noninferior to MoodGYM. The completion rates of IPT and CBT were higher than MoodGYM, indicating some progress in refining Internet-based self-help. Internet-delivered treatment options available for people suffering from depression now include IPT.

**Trial Registration:**

International Standard Randomized Controlled Trial Number (ISRCTN): 69603913; http://www.controlled-trials.com/ISRCTN69603913 (Archived by WebCite at http://www.webcitation.org/6FjMhmE1o)

## Introduction

Depression is a highly prevalent mental disorder [1] and it is expected to rank as the leading cause of burden of disease in high-income countries by 2030 [2]. Depression is associated with serious disability [3], loss in quality of life [4], and substantial economic costs both at an individual and a societal level [5,6]. Both pharmacological and psychological treatments for depressive disorders are effective in reducing symptoms [7]. Clinical practice guidelines recommend cognitive behavior therapy (CBT) and interpersonal psychotherapy (IPT) as options for psychological treatment [8-10]. CBT is based on the cognitive theory that negative automatic thoughts, maladaptive information processing, and avoidance behavior play a key role in the development and maintenance of depression [11]. IPT originates from interpersonal theory [12]. It links stressful life events and insufficient social support to the development and maintenance of depressive symptoms [13]. Both psychotherapies are brief, highly structured, and can be manualized. CBT and IPT have shown to be effective in reducing depression symptoms compared to treatment as usual [7,14,15]. Meta-analyses show that, when compared head-to-head, CBT and IPT do not differ significantly from one another in their effectiveness [7,14,15].

Both CBT and IPT require significant therapist time. Long waiting lists caused by low workforce numbers are common [16]. Perceived social stigma, which hinders help seeking [17], and high costs [18] may discourage individuals with a psychiatric disorder from seeking professional help. Internet-based self-help interventions offer potential solutions to these barriers. Immediately accessible and less costly, online interventions may offer a valuable alternative to face-to-face therapy. Previous studies and meta-analyses have demonstrated unguided Internet-based self-help interventions to be effective for common mental disorders, with a pooled effect size of 0.28, but dropout rates are high [8]. CBT programs have been successfully delivered on the Internet [19,20]. However, to our knowledge, no study has examined the effectiveness of Internet-based IPT.

The present study examined the effectiveness of Internet-delivered IPT and a new Internet-delivered CBT module (from e-couch [21]) compared to an online CBT intervention (MoodGYM). MoodGYM was originally developed for youth, but has known efficacy in reducing depressive symptoms in adults [22-24]. The trial was designed within a noninferiority framework. Noninferiority trials are used when there is clear evidence of efficacy for an existing standard treatment, such that it is ethically unacceptable to employ a placebo or inactive control group [25] and when a new treatment is hypothesized to have comparable, but not necessarily superior, effectiveness to the established intervention [26]. We hypothesized that the new Internet-delivered modules of IPT and CBT would be noninferior to a CBT module (MoodGYM) in reducing symptoms of depression and anxiety. We also predicted that the Internet-delivered IPT module would be rated by its users as being as feasible, acceptable, and satisfactory as MoodGYM.

## Method

### Participants and Procedure

This automated, 3-arm, fully self-guided, online noninferiority trial compared 2 new treatments (IPT and CBT) to an active control treatment (MoodGYM) for depressed individuals. The Internet-delivered CBT and IPT interventions (from e-couch) were developed at the Centre for Mental Health Research (CMHR) at the Australian National University (ANU). The e-couch program targets a range of conditions currently (depression, generalized anxiety disorder, social anxiety disorder) with other conditions to be added in the future. It also provides modules for bereavement and loss, as well as divorce and separation. It comprises a mental health literacy component and psychotherapeutic components for each condition (eg, CBT, IPT, applied relaxation, physical activity, and behavioral activation for depression). This study compared the IPT and CBT components with the 4-module version of MoodGYM. To increase external validity, there was no specific promotion for the trial. Spontaneous visitors from around the world who registered on the e-couch Internet website [21] between October 2009 and October 2010 and who showed interest in participating in the research trial (by clicking a “I want more information about the trial” button), were given information about the study. Those who provided both informed consent to participating in the trial (by clicking on the “I agree” button on the webpage) and an email address were then asked to complete an online baseline screening survey. Individuals who were 18 years of age or older and not currently receiving treatment for depression by a mental health specialist were eligible for inclusion in the study. Individuals with suicide intention, as measured with a suicidal ideation screening item on the Web Screening Questionnaire (WSQ) [27], or those who scored above 27 (95th percentile or higher) on the Center for Epidemiological Studies Depression scale (CES-D) at baseline, were immediately provided with an information page containing advice about obtaining appropriate professional help, including emergency help. They could, however, continue to participate in the study. Excluded were individuals who were health professionals treating people with depression or anxiety, researchers reviewing depression or anxiety sites, or students studying anxiety or depression as part of a college or university course. Individuals who did not meet the inclusion criteria were directed to the public version of the e-couch program, which provides interventions for depression, generalized anxiety disorder, and social phobia. Individuals were not required to provide their real names, but were asked to use a pseudonym instead. Ethical approval for the study was provided by the Human Research Ethics Committee of the ANU (protocol number 2008/269).

Participants were randomly assigned to MoodGYM, CBT, or IPT, stratified by sex, age, and presenting depression symptom severity. The randomization schedule for participant allocation to condition groups was prepared by using an automated system built into the trial software, and randomization occurred automatically. The allocation sequence was concealed from the researchers. Participants randomized to the intervention groups were aware of the allocated arm. Following randomization, an automated email containing log-in details for the assigned program was sent to each participant, at which point the intervention could be accessed immediately.

### Interventions

**Figure 1:**
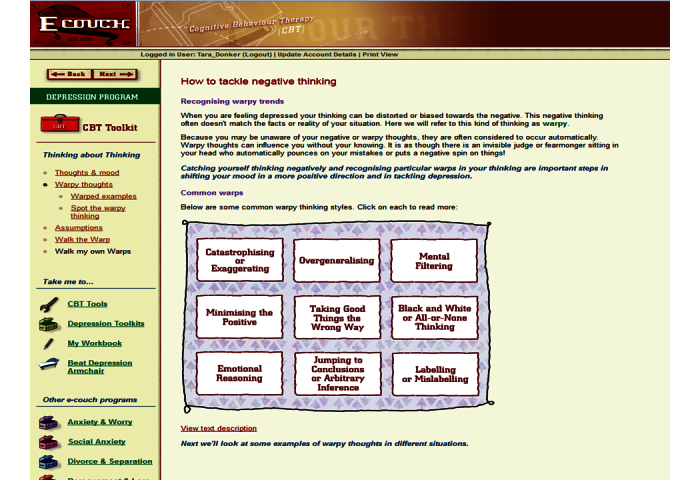
Screenshot of e-couch cognitive behavior therapy (CBT) website.

**Figure 2:**
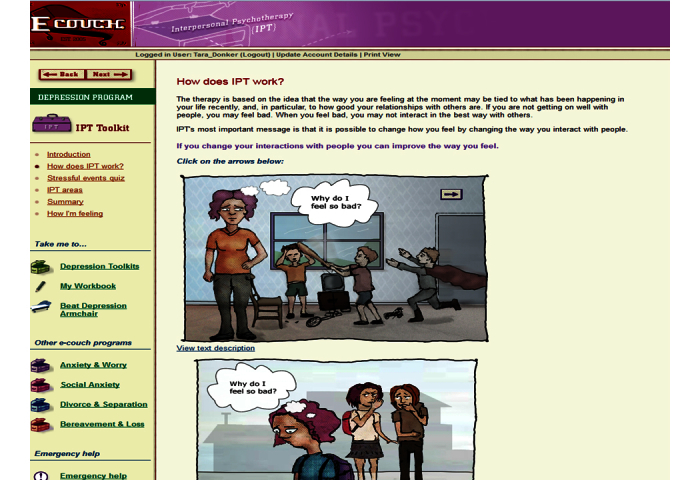
Screenshot of e-couch interpersonal psychotherapy (IPT) website.

**Figure 3:**
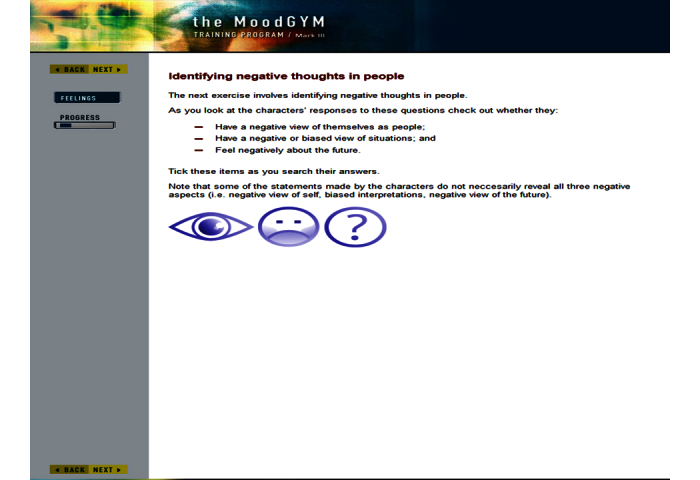
Screenshot of MoodGYM website.

All programs were offered over 4 weeks. Users were required to complete the modules in order. Participants were able to revisit previous pages of the modules and scores of previous assessments, but were not able to repeat the assessments. Each week an automated email was sent to advise participants of the availability of their new module. Participants were always offered the option to pause and restart at their chosen time. See Figures 1-3 for screenshots of the 3 programs.

### Internet-Delivered Cognitive Behavioral Therapy (CBT e-couch)

The Internet-delivered CBT intervention comprised 1 component of the depression stream of e-couch [21] and is based on the principles of CBT [11]. In addition to an explanation of the rationale of CBT, the program consisted of 3 major modules: identifying negative thoughts, tackling negative thoughts, and undertaking behavioral activation (based on activity scheduling developed by Lewinsohn [28]). The program contained 18 exercises and assessments in total, which were saved in a personal workbook.

### Internet-Delivered Interpersonal Psychotherapy (IPT e-couch)

The Internet-delivered form of IPT comprised 1 component of the depression stream of e-couch [21]. It consisted of 4 modules (grief, role disputes, role transition, and interpersonal deficits) and a personal workbook (containing 13 exercises and assessments). The IPT program was based on the IPT clinician manual of Weissman et al [13], with each of the 4 IPT areas constructed to reflect the areas and topics relevant to each area. Interactive exercises reflected the topics and questions described in the Interpersonal Inventory. Participants did not choose IPT areas, but could decide the order in which they were completed; exercises within each of the IPT areas were not compulsory.

### Internet-Delivered CBT (MoodGYM)

The online CBT package comprised a 4-module version of MoodGYM [29] delivered over 4 weeks. The details of the program are described elsewhere [30,31]. In this trial, a set of 4 of the CBT modules, a personal workbook (containing 22 exercises and assessments), and a feedback evaluation form were used. The modules cover the identification of and behavioral methods to overcome dysfunctional thinking, assertiveness, and self-esteem training. Each module takes approximately 20 to 40 minutes to complete [31]. The relaxation module was removed from the program for this study to match the time length of the other 2 programs. Previous research has demonstrated that this component is not needed for efficacy [31].

### Measures

All questionnaires comprised online standard self-report measures taken at baseline (pretest), immediately after the intervention (posttest), and 6 months after the intervention (follow-up). Measures of participant characteristics were collected at baseline, symptoms measures were administered at all 3 time points, and user satisfaction was collected at posttest.

### Participant Characteristics

The survey included questions concerning sociodemographic characteristics (age, gender, country of origin, location, and education level), previous history of depression, previous use of treatments for depression, marital status, preference for randomization condition, perceived need for treatment, and current medication.

### Primary Outcome Measures: Depressive Symptoms

#### Center for Epidemiological Studies Depression Scale

The 20-item self-report CES-D was used to assess depressive symptoms (item score: 0-3; total score range: 0-60) [32]. The Internet CES-D is reliable and valid with a cut-off score of 22 (sensitivity: 0.94; specificity: 0.62) [33]. The Cronbach alpha in this study was .90. Because the CES-D was administered online, a cut-off score of 22 is used in this study.

### Secondary Outcomes: Satisfaction, User-Perceived Benefits, and Adherence

#### Client Satisfaction Questionnaire

The Client Satisfaction Questionnaire (CSQ-8) assesses global client satisfaction with treatments [34]. The 8-item self-report questionnaire uses scale response options from 1 to 4, with total score ranges from 8 to 32. Previous research has reported that the CSQ-8 has high internal consistency [35] and was comparable to the Cronbach alpha in this study (Cronbach alpha=.90).

#### Treatment Preference

Preference for randomization condition was assessed by asking the question “Do you have a preference to be in one of the programs?” at baseline. Participants replied with no preference or “yes, program 1, 2, 3.” These data were included in the analyses (no preference, preference: match/no match).

#### Adherence

Adherence was measured in 2 ways for each individual: (1) completion of posttest surveys (all groups), and (2) the number of IPT, CBT, or MoodGYM modules completed.

### Statistical Analysis

**Figure 4:**
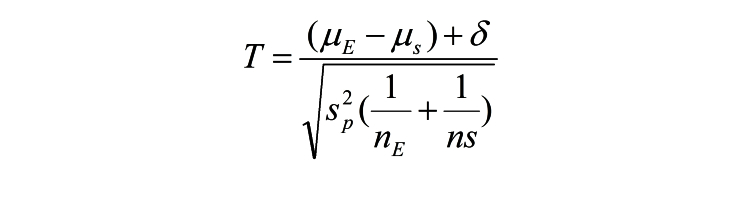
Formula of Mascha and Sessler.

Noninferiority trials require the a priori specification of a noninferiority margin. We used the confidence interval (CI) approach [36] to define the noninferiority margin for this study. The noninferiority margin of the primary outcome measure CES-D was set at a lower-bound 95% CI pre-post within-group effect size of 0.33, which was based on the lower-bound 95% CI margin of the pre-post within-group effect size of 0.56 (95% CI 0.33-0.79) of MoodGYM [24]. To decrease the risk of type 1 error (false acceptance of an ineffective treatment), the standard deviation (SD) of the pretest was used in calculating the lower-bound 95% CI margin of the effect size of 0.56; this yields a conservative estimate of benefit. For noninferiority trials, the null is E-S≤-delta and the alternative hypotheses (1-sided) is E-S>-delta, where *E* is the experimental treatment and *S* is the standard treatment. Figure 4 shows the formula for calculating the *t* test statistic when testing noninferiority (using the formula of Mascha and Sessler [26]).

The null hypothesis is rejected to claim noninferiority of the standard control treatment and the new treatment, if *T* is larger than the value of *T* from a *t* distribution with n_E_–n_S_–2df at 1–alpha. The *P* value is the probability of observing a larger value of *T*l, if the null hypothesis (ie, inferiority) were true in the population sampled from. For a *P* value less than alpha, we reject the null hypothesis and conclude noninferiority [26]. As this was a noninferiority trial, this criterion did not apply for the upper bound of the CI. Using 1-beta=.90 and alpha=.05 (2-sided), we needed at least 150 participants in each condition at posttest (a total sample of 450 participants) to reach sufficient statistical power.

Data integrity (distribution, outliers, skewness, and kurtosis) tests were conducted. Measures of skewness and kurtosis indicated deviations from normality for baseline CES-D scores because of some extreme responses. The Box-Cox model omitting the pretest scores was fitted and the transformed data were compared with the raw data using mixed model analyses. For ease of interpretation of the test results, only raw data are reported because the conclusions were the same. The baseline characteristics of the 3 groups were compared by using 1-way analysis of variance (ANOVA), and Kruskal-Wallis and Mann-Whitney U tests for continuous measures and chi-square (χ^2^) tests for categorical variables.

In noninferiority trials, intention-to-treat (ITT) analysis will often increase the risk of falsely claiming noninferiority (type I error) [37]. Non-ITT analyses are preferred as a protection from ITTs increase of type I error risk [38]. Therefore, the effects of the interventions were analyzed by using both ITT analyses and per-protocol approaches as recommended for noninferiority trials by Piaggio et al [38]. There is greater confidence in results when the conclusions are consistent [38].To conduct the per-protocol analysis, 2 groups were created: those who returned the posttest and follow-up surveys (completers), and those who completed half or more of the treatment modules and returned the surveys (adherent completers). Linear mixed models (LMM) were used for both types of analyses. Restricted maximum likelihood estimation was used with an unstructured covariance structure accommodation with participant effects. The LMM gives unbiased estimates of ITT effect under the assumption that data from participants who withdrew were missing at random (MAR). Test time was treated as a categorical variable because we were interested in the differences between groups on each occasion of measurement.

Between-group and within-group effect sizes were calculated according to Cohen’s *d* (standardized mean difference) [39]. Clinically meaningful changes on the CES-D were assessed using the clinically significant change (CSC) formula (with a CES-D score <22) and the reliable change index (RCI) [40]. The RCI reflects the degree of change that occurred beyond the fluctuations of an imprecise measuring instrument, with values greater than 1.96 representing statistically significant change [41]. In the present study, pretest SD scores of the CES-D with a reliability of 0.90 were used in the RCI formula. In addition, analyses were also undertaken for the subsample of participants who had symptoms severe enough to be considered clinical cases at baseline (score ≥22 on the CES-D). Chi-square tests and 1-way ANOVA tests with Bonferroni correction at posttest were used to examine differences in treatment completers and noncompleters (those who completed less than half of the modules). Statistical analyses were conducted using SPSS version 19.0, except for the Box-Cox transformation procedures, which were conducted using Stata 9.

## Results

### Participants

**Figure 5:**
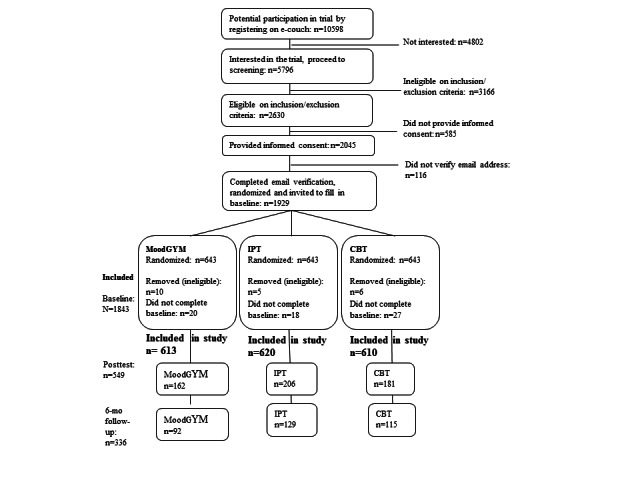
Flowchart of participants.

Of the 10,598 individuals who registered on e-couch during the trial period, 5796 expressed interest in the trial and proceeded to screening. Of these, 3166 did not meet the eligibility criteria (eg, under 18 years of age or currently receiving treatment for depression by a mental health specialist) and were excluded from the study. Of the remaining 2630 participants, 2045 provided informed consent; however, 116 of these did not subsequently verify their email address. Accordingly, a total of 1929 participants were randomized to 1 of the 3 conditions. However, 66 of these participants were excluded after randomization because it became apparent that they were ineligible at baseline for participation (eg, being a researcher or a student, n=21). In addition, 45 randomized participants did not complete the baseline assessment. This was missed at first screening because of a technical fault, but was picked up subsequently. Figure 5 shows the flowchart of participants (CONSORT-EHEALTH checklist [42] presented in Multimedia Appendix 1).

Of the total sample (N=1843), 543 (29.46%) were aged between 25 to 29 years, and most were female, (1334/1843, 72.38%). Participants were primarily Australian or New Zealand residents (751/1843, 40.75%) and most were well educated, having completed postsecondary education (1606/1843, 87.14%). The mean CES-D baseline score was 36 (SD 11.52). There were no significant differences between the groups at baseline with respect to depressive symptoms (χ^2^_2_= 3.1, *P*=.21), demographic characteristics (see Table 1), or treatment preference before randomization (*P*=.73).

### Treatment Adherence and Attrition

In total, 30% (549/1843) of participants completed the posttest assessment and 28% (336/1843) completed the follow-up assessment. Of participants who were adherent to the program (completed 50% or more of the modules), 25.8% (476/1843) and 16% (294/1843) completed posttest and follow-up assessments, respectively. Of the IPT participants, 49.5% (307/620) completed at least half of the intervention (≥2 modules) and 27.3% (169/620) completed all modules. For the CBT participants, 37.7% (230/610) finished 2 or more modules and 14.4% (88/610) completed all modules. A total of 195 of 613 participants (31.8%) finished half or more of the MoodGYM program. Of these, 10.9% (67/613) finished the whole program. Reasons given for dropout included technical problems, personal issues (lack of time), disease-specific barriers (feeling too depressed to work on the program or not convinced that the program would help), general intervention problems (programs was taking too long, too much text to read, boring, or too repetitive), specific intervention issues (the examples were not relevant to the participant), or engagement issues (preferred to obtain help from somewhere other than a computer). However, most participants (1248/1294, 96%) did not provide any reason for dropout. Those who dropped out of treatment had significantly higher scores on the CES-D (χ^2^_1_=4.3, *P*=.04), but differences were small (mean difference 1.26). Furthermore, dropout rates were significantly higher for participants assigned to MoodGYM (451/613, 74%) compared with IPT (414/620, 67%) or CBT (429/610, 70%; χ^2^_2_= 6.8, *P*=.03). Those who dropped out of treatment were more likely to be female (914/1294, 71%; χ^2^_1_= 6.6, *P*=.01), and under 50 years of age (1090/1294, 84%; χ^2^_1_=21.6, *P*<.001). No significant differences were found for medication use (*P*=.73), treatment preference (*P*=.14), or marital status (*P*=.60).

### Effectiveness and Noninferiority

Results are presented for 3 groups: all participants (all those enrolled in the trial, ITT), completers (those completing online surveys at posttest and at 6-month follow-up), and adherent completers (those completing ≥50% of the modules).

For completers, the within-group effect sizes on the primary outcome measure CES-D were large for all treatments at posttest (IPT *d*=0.76 vs CBT *d*=0.87) and follow-up (IPT *d*=1.02 vs CBT *d*=1.44). Between-group effect sizes were small (posttest: IPT vs MoodGYM *d*=0.14, 95% CI –0.06 to 0.35; CBT vs MoodGYM *d*=0.05, 95% CI –0.17 to 0.26; follow-up: IPT vs MoodGYM *d*=0.18, 95% CI –0.09 to 0.45; CBT vs MoodGYM *d*=0.12, 95% CI –0.15 to 0.39). Within-group effect sizes for adherent completers ranged from *d*=0.74 to *d*=0.90 at posttest and *d*=1.02 to *d*=1.33 at follow-up. The between-group effect size for IPT vs MoodGYM was higher (posttest: *d*=0.23, 95% CI 0.0-0.46; follow-up: *d*=0.31, 95% CI 0.02-0.60) than that for CBT vs MoodGYM (posttest: *d*=0.02, 95% CI –0.25 to 0.22; follow-up: *d*=0.04, 95% CI –0.26 to 0.34). The ITT analyses yielded medium within-group effect sizes (*d*=0.59 to *d*=0.67 at posttest and *d*=0.66 to *d*=0.80 at follow-up). Between-group effect sizes were small (posttest: IPT vs MoodGYM *d*=0.09, 95% CI–0.02 to 0.21); CBT vs MoodGYM *d*=0.01, 95% CI –0.10 to 0.12; follow-up: IPT vs MoodGYM *d*=0.09, 95% CI –0.02 to 0.21; CBT vs MoodGYM *d*=0.03, 95% CI –0.08 to 0.14). See Table 2 and Multimedia Appendix 2.

The previously determined noninferiority margin (*d*=0.33) was converted to delta=3.795 points differences on the CES-D (based on a SD of 11.5). Using the formula of Mascha and Sessler [26], a completers analysis indicated that IPT compared to MoodGYM was found to be noninferior at posttest (*t*_366_=4.046, *P*<.001, 95% CI –0.89 to 4.73). The mean difference between IPT and MoodGYM on the CES-D for completers at posttest was 1.92 points (95% CI –0.86 to 4.70, *P*=.17). MoodGYM participants scored nonsignificantly lower at posttest. CBT completers were also found to be noninferior to MoodGYM (*t*_341_=2.142, *P*=.02; 95% CI –3.57 to 2.33), with a mean difference at posttest of 0.62 points (lower for CBT) which was not statistically significant (95% CI –2.30 to 3.54, *P*=.68). For adherent completers, results were similar (IPT vs MoodGYM posttest: *t*_316_=4.506, *P*<.001; CBT vs MoodGYM posttest: *t*_282_=2.246, *P*<.001; 95% CI –3.39 to 2.91). There was a nonsignificant mean difference between IPT and MoodGYM on the CES-D for adherent completers at posttest of 3.05 which was higher for IPT (95% CI 0.06-6.04, *P*=.05), but not for CBT vs MoodGYM (mean difference: 0.24; 95% CI –2.88 to 3.36, *P*=.88, lower for CBT). An ITT analysis also indicated that IPT and CBT were found to be noninferior to MoodGYM (IPT: *t*_1231_=4.769, *P*<.001, 95% CI –0.41 to 4.43; CBT: *t*_1221_=3.207, *P*<.001, 95% CI –2.27 to 2.71). Mean depression scores were not significantly different across the 3 programs at posttest (IPT vs MoodGYM: 2.01, 95% CI –0.32 to 4.34, *P*=.09, higher for IPT; CBT vs MoodGYM: 0.22, 95% CI: –2.17 to 2.61, *P*=.86, higher for CBT).

Table 2 presents the means and SDs for completers, adherent completers, and the ITT sample as produced by the LMM procedure. Because LMM does not yield SDs, we calculated them manually by using the formula SD=SEM×√N. For the completers of posttest and/or follow-up, there was a significant overall improvement over time for all groups on the CES-D (*F*_2,434.0_=290.309, *P*<.001). There was no significant group×time interaction effects on the CES-D at posttest (*F*_4,436.3_=1.15, *P*=.33). Results were similar for the ITT sample and the adherent completers (see Table 3).

Residuals of the models were inspected and showed nonnormality. Therefore, to be thorough, power transforms were estimated fitted using a Box-Cox model that included the same terms as the mixed model omitting the pretest scores. The test of deviations of residuals from normality was significant for just the IPT group at posttest (*t*_386.273_=2.36, *P*=.02). We compared the contrast results by using the transformed data to the raw data, indicated that they have the same pattern of significance. Because the normality violation was not profound and because it is easier to interpret raw data (and retransformation of model estimates is not always appropriate), we have presented raw data in this paper.

**Table 1:** Baseline demographic, socioeconomic, and clinical characteristics of participants for the e-couch cognitive behavioral therapy (CBT), the e-couch interpersonal psychotherapy (IPT), and the MoodGYM website.

Condition		All participants	MoodGYM	CBT	IPT
n/N (%)		1843 (100)	613 (33.26)	610 (33.10)	620 (33.64)
Female, n (%)		1334 (72.38)	438 (71.45)	445 (72.95)	451 (72.74)
**Age group (years), n (%)**					
	18-24	307 (16.66)	100 (16.31)	92 (15.08)	115 (18.55)
	25-34	543 (29.46)	181 (29.52)	188 (30.82)	174 (28.06)
	35-44	470 (25.50)	145 (23.65)	164 (26.88)	161 (25.97)
	45-55	338 (18.34)	111 (18.11)	113 (18.52)	114 (18.39)
	>55	185 (10.04)	76 (12.39)	53 (8.69)	56 (9.03)
**Country of residence, n (%)**					
	Australia and New Zealand	751 (40.75)	254 (41.44)	239 (39.18)	258 (41.61)
	United Kingdom	454 (24.63)	148 (24.14)	157 (25.73)	149 (24.03)
	United States	350 (18.99)	112 (18.27)	115 (18.85)	123 (19.84)
	Canada	100 (5.43)	28 (4.57)	36 (5.90)	36 (5.81)
	Other	188 (10.20)	71 (11.58)	63 (10.32)	54 (8.71)
Spouse		914 (49.59)	301 (49.10)	310 (50.82)	303 (48.87)
**Education**					
	None, or primary	21 (1.13)	11 (1.79)	4 (0.66)	6 (0.97)
	Secondary	216 (11.72)	70 (11.42)	67 (10.98)	79 (12.74)
	Postsecondary	1606 (87.14)	532 (86.79)	539 (88.36)	535 (86.29)
Baseline CES-D^a^, mean (SD)		36.01 (11.52)	35.34 (11.61)	36.29 (11.04)	36.38 (11.86)
Current medication^b^, n (%)		754 (40.91)	253 (41.27)	255 (41.80)	246 (39.68)

^a^CES-D: Center for Epidemiological Studies Depression scale.

^b^Any prescribed current medication.

**Table 2:** Results and effect sizes (Cohen’s *d*) for the Center for Epidemiological Studies Depression scale (CES-D) for completers and adherent completers, and for intention-to-treat (ITT) analyses.

Program		Test time, n; mean (SD)			Within-group effect size, *d* (95% CI)		Between-group effect size, *d* (95% CI)			
		Pretest	Posttest	Follow-up	Pre-post	Pre–follow-up^a^	Program		Posttest	Follow-up (95% CI)
**Completers of posttest (n=549) and/or follow-up (n=336)**										
	IPT	206; 35.65 (11.85)	206; 26.22 (12.92)	129; 22.41 (13.84)	0.76 (0.56,0.96)	1.02 (0.76,1.28)	IPT vs MoodGYM		0.14 (–0.06,0.35)^b^	0.18 (–0.09,0.45)^b^
	CBT	181; 34.46 (11.31)	181; 23.68 (13.34)	115; 18.17 (12.15)	0.87 (0.65,1.0 9)	1.44 (1.15,1.72)	CBT vs MoodGYM		0.05 (–0.17,0.26)^c^	0.12 (–0.15,0.39)^c^
	MoodGYM	162; 35.19 (12.44)	162; 24.30 (14.10)	92; 19.79 (14.92)	0.82 (0.59,1.04)	1.04 (0.72,1.34)				
**Adherent completers of posttest (n=476) and/or follow-up (n=294)**										
	IPT	192; 35.60 (11.79)	192; 26.38 (13.25)	119; 22.50 (13.55)	0.74 (0.53,0.94)	1.02 (0.74,1.28)	IPT vs MoodGYM	0.23 (0.0,0.46)^b^		0.31 (0.02,0.60)^b^
	CBT	158; 34.30 (11.79)	158; 23.09 (13.25)	101; 17.75 (13.55)	0.89 (0.65,1.11)	1.33 (1.02,1.63)	CBT vs MoodGYM	0.02 (–0.25,0.22)^c^		0.04 (–0.26,0.34)^c^
	MoodGYM	126; 34.41 (11.32)	126; 23.33 (13.25)	74; 18.30 (13.55)	0.90 (0.64,1.16)	1.21 (0.86,1.56)				
**ITT posttest and follow-up (N=1843)**										
	IPT	620; 36.38 (11.51)	620; 26.59 (20.27)	620; 23.17 (25.60)	0.59 (0.48,0.71)	0.67 (0.55,0.78)	IPT vs MoodGYM	0.09 (–0.02,0.21)^b^		0.09 (–0.02,0.21)^b^
	CBT	610; 36.29 (11.51)	610; 24.80 (21.34)	610; 19.68 (26.85)	0.67 (0.55,0.79)	0.80 (0.69,0.92)	CBT vs MoodGYM	0.01 (–0.10,0.12)^b^		0.03 (–0.08,0.14)^c^
	MoodGYM	613; 35.34 (11.52)	613; 24.58 (22.43)	613; 20.56 (29.69)	0.60 (0.49,0.72)	0.66 (0.54,0.77)				

^a^Within-group follow-up effect size for completers is based upon the following pretest scores: IPT (n=129, mean 35.66, SD 12.05); CBT (n=115, mean 34.89, SD 11.05); MoodGYM (n=92, mean 34.13, SD 12.65); within-group follow-up effect size for adherent completers is based upon the following pretest scores: IPT (n=119, mean 35.48, SD 11.91); CBT (n=101, mean 34.68, SD 11.90); MoodGYM (n=74, mean 33.77, SD 11.92).

^b^In favor of MoodGYM.

^c^In favor of CBT.

**Table 3:** Effectiveness of Internet-delivered programs with depression score (CES-D) as dependent variable.

Depression score		Posttest				Follow-up			
		Time		Group×time		Time		Group×time	
		*F* (df)	*P*	*F* (df)	*P*	*F* (df)	*P*	*F* (df)	*P*
**Total sample**									
	Completers	290.309 (2,434.0)	<.001	1.15 (4,436.3)	.33	237.187 (2,315.1)	<.001	1.20 (4,315.3)	.31
	Adherent completers	260.021 (2,386.7)	<.001	1.52 (4,388.3)	.20	216.083 (2,284.1)	<.001	1.426 (4,284.5)	.23
	Intention-to-treat	382.60 (2,484.155)	<.001	1.45 (4,483.246)	.22		<.001		
**Clinical cases**									
	Completers	306.190 (2,368.8)	<.001	.976 (4,369.4)	.42	223.572 (2,242)	<.001	0.824 (4,242)	.51
	Adherent completers	275.800 (2,327.7)	<.001	1.39 (4,328.3)	.24	230.990 (2,242.9)	<.001	1.056 (4,243.1)	.38
	Intention-to-treat	306.190 (2,368.8)	<.001	0.976 (4,451.2)	.42				

### Clinically Significant Change and Reliable Change Index for Completers

For completers, no significant differences in CSC were found between the 3 programs at posttest (χ^2^_2_=1.78, *Ρ*=.41) and follow-up (χ^2^_2_=3.70, *P*=.16). The number of participants showing CSC at posttest were n=61 for IPT, n=65 for CBT, and n=52 for MoodGYM. For adherent completers, results were similar. Using the formula of Jacobson and Truax [40] for RCI (the degree of change that occurred beyond the fluctuations of an imprecise measuring instrument) with a Cronbach alpha of .90, all programs reached the RCI critical value of 1.96 at posttest (IPT: 2.01; CBT: 2.41; MoodGYM: 2.21) and follow-up (IPT: 2.78; CBT: 3.82; MoodGYM: 2.86). The RCI results were similar for adherent completers at posttest (IPT: 1.97; CBT: 2.40; MoodGYM: 2.47) and follow-up (IPT: 2.76; CBT: 3.59; MoodGYM: 3.28; see Table 4).

### Clinical Cases

Analyses were also undertaken for the subsample of participants who had symptoms severe enough to be considered clinical cases at baseline. A CES-D value of ≥22 is considered to indicate clinical caseness [31]. For the ITT sample scoring ≥22 on the CES-D, analyses showed a significant overall improvement over time for all groups on the CES-D (*F*_2,368.8_=306.190, *P*<.001). No differences between the treatments over time were found on the CES-D (*F*_4,451.2_=0.976, *P*=.42). Within-group effect sizes on the primary outcome measure CES-D were small for all treatments at posttest (IPT *d*=0.55; CBT and MoodGYM *d*=0.56) and medium at follow-up (CBT *d*=0.65; MoodGYM *d*=0.61) except for IPT, which was small (IPT *d*=0.58). The RCI was below the critical value of 1.96 at posttest for all programs. At follow-up, all programs reached the critical value of 1.96 (IPT: 2.26; CBT: 2.51; MoodGYM: 2.46). For completers and adherent completers scoring ≥22 on the baseline CES-D, results were similar (see Table 3). RCI for completers was above 1.96 for all programs at posttest (IPT: 2.93; CBT: 3.26; MoodGYM: 3.47) and follow-up (IPT: 3.84; CBT: 4.80; MoodGYM: 4.97). RCI for adherent completers was above 1.96 for all programs at posttest (IPT: 2.95; CBT: 3.40; MoodGYM: 3.57) and follow-up (IPT: 4.00; CBT: 4.93; MoodGYM: 4.77, see Multimedia Appendix 3).

### Treatment Satisfaction

There was a significant difference between the 3 interventions in treatment satisfaction scores at posttest for completers as measured with the CSQ-8 (*F*_2,535_=18.75, *P*<.001). Post hoc analyses using Tukey’s honestly significant difference (HSD) test showed that participants randomized to IPT (n=201) had a significantly lower total satisfaction score (mean 20.55, SD 4.80) compared to MoodGYM (n=158; mean 22.81, SD 4.58) with a mean difference of 2.26 (SD 0.49, *P*<.001) and CBT (n=179; mean 23.26, SD 4.47) with a mean difference of 2.71 (SD 0.48, *P*<.001).

**Table 4:** Proportion of participants reaching the criteria for clinically significant change (score <22) on the Center for Epidemiological Studies Depression scale (CES-D).

Treatment condition	Baseline caseness, n (%)	Clinically significant change			
		Posttest n (%)		6-month follow-up n (%)	
		Completers^a^	Adherent completers^b^	Completers^c^	Adherent completers^d^
IPT (n=610)	581 (95.2)	61 (32.0)	55 (32.7)	54 (43.5)	49 (48.0)
CBT (n=620)	581 (93.7)	65 (38.2)	61 (43.6)	63 (57.3)	32 (36.0)
MoodGYM (n=613)	575 (93.8)	52 (34.7)	41 (39.4)	42 (51.2)	36 (59.0)

^a^Completers posttest IPT (n=194), CBT (n=170), MoodGYM (n=150).

^b^Adherent completers posttest IPT (n=168), CBT (n=140), MoodGYM (n=104).

^c^Completers 6-month follow-up IPT (n=124), CBT (n=110), MoodGYM (n=82).

^d^Adherent completers IPT (n=102), CBT, MoodGYM (n=61).

## Discussion

### Principal Results: Noninferiority, Effectiveness, and Efficacy

The present study is the first to show that Internet-delivered IPT can be effective in the treatment of depressive symptoms at posttest and at 6-month follow-up. Both the IPT and the CBT online interventions employed in the trial showed significant medium to large within-group effect sizes on the CES-D for completers and adherent completers. For the ITT sample, effect sizes were smaller, but still moderate in size. Of the clinical cases, completers and adherent completers showed medium to large effect sizes on posttest and follow-up ratings. We found that IPT and CBT were noninferior compared to MoodGYM for those who returned posttest, and between-group effect sizes were small. Although recent MoodGYM studies report similar effect sizes to our study, our conclusions need to be taken with some caution given that the effect size found in this study differed from the effect size from the initial study, and therefore might hamper assay sensitivity. Furthermore, the new CBT program reached consistently higher, but not significant, effect sizes compared to the IPT and the standard MoodGYM program. Overall, the between-group effect sizes were larger for IPT versus MoodGYM compared to CBT versus MoodGYM.

### Comparison With Prior Work

Our findings of the equivalent effectiveness of CBT and IPT are concordant with previous research on face-to-face interventions [7]. The within-group effect size for completers of MoodGYM found in our study was similar to that of 2 recently published studies of unguided MoodGYM [23,43], but was considerably higher than the trial conducted by its originators in 2004 [22,24]. To be able to draw reliable conclusions of noninferiority, it is important to establish effect sizes of similar size to prior trials. To minimize bias, it is important to replicate the conditions under which the control treatment was previously examined (eg, the same population sample, outcome measures, assessment time points, and delivery of treatment). MoodGYM is automated and has fidelity as an intervention. We have no reason to assume that omission of the MoodGYM relaxation module accounts for the difference in the effect size found in this study, because removal did not affect treatment effectiveness in a previous dismantling study [31]. The most likely cause of the observed differences lies in the different samples recruited. The present study recruited participants directly from those visiting a self-help website, whereas the original study consisted of a sample of participants selected randomly from the Australian electoral roll. The present sample had higher depression scores at baseline compared to the original trial [22,24]. The difference in dropout rate (42% in the original study vs 70% in our study) might also account for the higher effect size we found for our completer analysis, because the effect size in our ITT sample (MoodGYM *d*=0.66) is more similar to the effect size of the completers in the originators study (MoodGYM *d*=0.56) [22,24]. Lower dropout rates in the original study may have arisen from the addition of weekly phone calls by lay interviewers, which might be considered as minimal contact therapy [44] and might affect the dropout rate [45]. However, a recent study by Farrer et al [23] found no significant difference in dropout between participants receiving weekly telephone calls in addition to MoodGYM and those receiving only self-guided MoodGYM. This aside, we can conclude that Internet-delivered IPT is likely to be an effective treatment for depressive symptoms, and thereby offers people with depression another online treatment option

The CSC rates in the current study were lower than those reported in other online studies [46,47]. One explanation for this finding might be that those studies incorporated guidance, whereas this study was fully automated. Another explanation might be that the baseline CES-D scores were higher in this study than typically found in other studies [47]. Hence, the drop in CES-D score required to achieve a score in the nonclinical range (ie, a CSC) is more difficult to reach. Clinical cases of the ITT samples showed no reliable change. However, completers of clinical cases showed reliable change for all programs at posttest and follow-up. CBT reached consistently higher RCI rates. CBT might be superior to IPT and/or MoodGYM. However, as we did not set our hypothesis a priori to test noninferiority between IPT and CBT, or superiority between CBT and the other programs, conclusions cannot be drawn because of insufficient power.

### Feasibility and Satisfaction

There was no significant difference in treatment preference at baseline before randomization. This lack of preference for treatment condition is important, because it suggests that a disparity between the preferred and allocated conditions was unlikely to negatively impact disproportionately on the findings. However, treatment satisfaction ratings were significantly lower for the IPT program compared with MoodGYM and CBT. One explanation for these findings may be related to what people were looking for in an online intervention. Also, although there were no patient program preferences before randomization, it is unknown how participants felt about being randomized to IPT immediately postrandomization but before exposure to the treatment. Online CBT is widely known, whereas fewer individuals know about IPT. To the extent that the social psychological literature has demonstrated that familiarity breeds liking, it may be possible that differences in satisfaction in treatment were driven in part by differences in familiarity with each treatment. Another explanation may be that the IPT program was too brief. Adherence to the treatment was considerably lower than the original MoodGYM trial [22]. As mentioned earlier, the influence of weekly telephone calls in the original study might have influenced the dropout rate, as might the source of participants from among spontaneous visitors to a self-help website. Some studies of unguided self-help have reported similar dropout rates [43,48,49], but others have not [7,50]. Completion rates for the new IPT and CBT programs were significantly higher than for MoodGYM. This could suggest that the new programs are more acceptable, particularly to adults. Within a noninferiority framework, this finding is very important, because newer implementations of e-therapy were at least as effective as MoodGYM, whereas completion rates—a key problem in this field [51]—were higher. However, MoodGYM is a well-known open-access program. Some participants assigned to MoodGYM could have undertaken the program previously, and if so may have been less willing to finish the intervention.

### Limitations

This study has several limitations. First, as mentioned previously, the effect size found in this study differs from that on which we based the noninferiority margin and power calculation. Second, the noninferiority margin of the primary outcome measure is usually based on the lower-bound CI of the between-group effect size of the traditional treatment [36]. In our case, this would be an effect size of 0.33 and a lower-bound 95% CI of 0.11 [24]. To reach sufficient statistical power to be able to detect a significant difference, we would need at least 14,000 participants per condition. Therefore, we used an alternative approach to calculate the lower-bound noninferiority margin. Based on the study of Mackinnon et al [24] we used the within-group effect size of 0.56 instead. This resulted in a noninferiority margin of an effect size of 0.33, which is a 3.795 difference on the CES-D. Although this difference is still liberal, an effect size of 0.30 is considered as the minimum for clinically meaningful change [52]. Third, LMM is based on the MAR assumption, while dropout rates were very high. It is widely recognized that the MAR assumption is untestable. MAR assumes that the pattern of missing data does not depend on the unobserved data. This is a substantially weaker assumption than missing completely at random (MCAR), in which the data are missing independent of values of the observed and unobserved data. Therefore, LMM is the most robust of the methods for analyzing the data.

Low adherence, however, could underestimate differences between groups, and therefore increase the likelihood of finding noninferiority. However, our completer analyses revealed no statistical differences in effectiveness between the 3 programs. Nevertheless, our conclusions need to be taken with caution given the high dropout rates. One possible explanation of the difference in attrition rates across the programs might be that MoodGYM takes longer to complete compared with the other programs, and lengthier programs might be associated with greater attrition [31]. Although MoodGYM had the highest dropout rate, dropout was high among all conditions, a finding that is common for Internet interventions. High dropout rates are likely with minimal exclusion criteria, unguided interventions [44,45], and little or no financial commitment [51]. However, a recent study by Hilvert-Bruce et al [53] showed that noncompleters derive benefit before dropping out. Also, there were significant baseline differences (CES-D score, gender, age) between participants who completed the programs and those who did not, which might indicate selection bias. The ITT analyses demonstrated effects nevertheless. In addition, it was unknown whether participants used other treatments during the study. This could mask real differences between groups if this use of additional treatments was more prevalent for 1 group compared with the others. Because we did not measure additional use of other treatments, we cannot rule this possibility out entirely. Finally, in face-to-face IPT, one focus is chosen, whereas in the Internet-delivered IPT, all modules were undertaken by the participant.

Future research is needed to replicate IPT noninferiority compared to CBT programs, to test whether the new CBT program is superior to other programs, to examine whether guided Internet-delivered IPT is as effective as face-to-face IPT, to investigate methods to improve adherence, to investigate whether IPT would also be effective outside of a randomized controlled trial setting, and whether Internet-delivered IPT is also effective in the treatment of other disorders, such as social phobia or panic disorder. It is important that future research investigates individual characteristics, such as recent life events, that predict treatment response for IPT. There will also be value in investigating whether a planned extended version of e-couch IPT will yield higher satisfaction ratings.

### Conclusions

Although a firm conclusion regarding the noninferiority of IPT and the sustainability of results compared to CBT cannot be drawn yet, we can conclude that Internet-delivered IPT is an effective treatment for depressive symptoms, and thereby offers those with depression another online treatment option. An Internet-accessed IPT program could potentially be more appealing to IPT-trained therapists than a CBT-based one, perhaps making such clinicians more likely to recommend it to their clients. In the United Kingdom, the first wave of the Increasing Access to Psychological Therapies (IAPT) initiative was CBT only, but recently it has been expanded to other approaches, including IPT. Given that MoodGYM is already a resource used within IAPT (mostly without support), Internet-delivered IPT could well be a feasible option in second-wave IAPT services. Furthermore, the new e-couch CBT program was shown to be noninferior to the active CBT-based control program and thus may provide an open-access alternative to MoodGYM. Another important finding is that the completion rates of the new treatments were higher, indicating some progress in refining Internet-based self-help.

## Multimedia Appendix 1

CONSORT-EHEALTH checklist V1.6.2 [42].

## Multimedia Appendix 2

Mean depression scores over time for interpersonal psychotherapy (IPT), cognitive behavior therapy (CBT), and MoodGYM (intention-to-treat, N=1843).

## Multimedia Appendix 3

Results and effect sizes (Cohen's *d*) for Center for Epidemiological Studies Depression scale (CES-D) per protocol and intention-to-treat (ITT) analyses for clinical cases (≥22 on the CES-D).

## Abbreviations

ANU: The Australian National University
CBT: cognitive behavioral therapy
CES-D: Center for Epidemiological Studies Depression scale
CMHR: Centre for Mental Health Research
CSC: clinically significant change
CSQ-8: Client Satisfaction Questionnaire
IAPT: Increasing Access to Psychological Therapies
IPT: interpersonal psychotherapy
ITT: intention-to-treat
LMM: linear mixed model
MAR: missing at random
RCI: reliable change index
WSQ: Web Screening Questionnaire
