# Supplementary material for: Internet-Delivered Interpersonal Psychotherapy Versus Internet-Delivered Cognitive Behavioral Therapy for Adults With Depressive Symptoms: Randomized Controlled Noninferiority Trial
Source: J Med Internet Res. 2013 May 13;15(5):e82. doi: 10.2196/jmir.2307 (PMC3668608; doi:10.2196/jmir.2307)
Supplement: Supplementary file 1 [file jmir_v15i5e82_app1.pdf]

# CONSORT-EHEALTH Checklist V1.6.2 Report

**(based on CONSORT-EHEALTH V1.6), available at [http://tinyurl.com/consort-ehealth-v1-6].**

2307

**Date completed**
8/15/2012 10:44:13
**by**
Tara Donker

Internet-delivered Interpersonal Therapy versus internet-delivered Cognitive Behaviour Therapy for adults with depressive symptoms: A Randomized Controlled Noninferiority Trial

## TITLE

**1a-i) Identify the mode of delivery in the title**
we have used: "Internet-delivered".
**1a-ii) Non-web-based components or important co-interventions in title**
The interventions are completely online, there are no offline components.
**1a-iii) Primary condition or target group in the title**
"adults with depressive symptoms"

## ABSTRACT

**1b-i) Key features/functionalities/components of the intervention and comparator in the METHODS section of the ABSTRACT**
"An automated, three-arm, fully self-guided online noninferiority trial compared two new treatments (IPT [n=620] and CBT [n=610]) to an active control treatment (MoodGYM, n=613) over a four week period in the general population."
**1b-ii) Level of human involvement in the METHODS section of the ABSTRACT**
There was no human contact whatsoever. "An automated, three-arm, fully self-guided online noninferiority trial compared two new treatments (IPT [n=620] and CBT [n=610]) to an active control treatment (MoodGYM, n=613) over a four week period in the general population. Outcomes were assessed using online self-report questionnaires (CES-D and CSQ-8) completed immediately following treatment and at 6-month follow-up."
**1b-iii) Open vs. closed, web-based (self-assessment) vs. face-to-face assessments in the METHODS section of the ABSTRACT**
participants were recruited online, this is a purely web-based trial, outcomes were self-assessed through questionnaires (online).

"This study examines whether IPT is effective, noninferior to, and as feasible as CBT when delivered online to spontaneous visitors of an online therapy website."

"Outcomes were assessed using online self-report questionnaires (CES-D and CSQ-8) completed immediately following treatment and at 6-month follow-up."
**1b-iv) RESULTS section in abstract must contain use data**
No. of participants: (IPT [n=620] and CBT [n=610]) to an active control treatment (MoodGYM, n=613)"

primary outcomes:
"Completers-analyses showed a significant reduction in depressive symptoms at post-test and follow-up for both CBT and IPT, and were noninferior to MoodGYM. Within-group effect sizes were medium to large for all groups. treatments There were no differences in Clinical Significant Change between the programs. Reliable Change was shown at post-test and follow-up for all programs, with consistently higher rates for CBT.

secondary outcome: "Participants allocated to IPT showed significantly lower treatment satisfaction compared to CBT and MoodGYM."

adherence: "There was a drop-out rate of 70% at post-test, which was highest for MoodGYM. "
**1b-v) CONCLUSIONS/DISCUSSION in abstract for negative trials**
"Despite a high drop-out rate and lower satisfaction scores, this study suggests that Internet-delivered self-guided IPT is effective in reducing depressive symptoms, and may be non-inferior to MoodGYM. The completion rates of IPT and CBT were higher than MoodGYM, indicating some progress in refining internet-based self-help. Internet-delivered treatment options available for people suffering from depression now include IPT."

## INTRODUCTION

**2a-i) Problem and the type of system/solution**
Problem: "Both CBT and IPT require significant therapist time. Long waiting lists caused by low workforce numbers [17] (Cameron and Thompson, 2005) are common. Perceived social stigma, which hinders help seeking [18] (Barney et al., 2006), and high costs (Palmvqist et al., 2007) [19] may discourage individuals with a psychiatric disorder from seeking professional help. Internet-based self-help interventions offer potential solutions to these barriers."
problem:

Solution: "Immediately accessible and less costly, online interventions may offer a valuable alternative to face-to-face therapy"

Problem: "no study has examined the effectiveness of internet-based IPT."

aim: "The present study examined the effectiveness of internet-delivered IPT and a new internet-delivered CBT module (from e-couch; www.ecouch.anu.edu.au) compared to an online CBT intervention (MoodGYM"
**2a-ii) Scientific background, rationale: What is known about the (type of) system**
"Meta-analyses show that, when compared head-to-head, CBT and IPT do not differ significantly from each other in their effectiveness"

"Previous studies and meta-analyses have demonstrated unguided internet-based self-help interventions to be effective for common mental disorders, with a pooled effect size of 0.28, but drop-out rates are high [9] (Cuijpers et al., 2011a).  CBT programs have been successfully delivered on the Internet [20,21] (Andrews et al., 2010; Griffiths et al., 2010). "

## METHODS

**3a) CONSORT: Description of trial design (such as parallel, factorial) including allocation ratio**

objective: " The present study examined the effectiveness of internet-delivered IPT and a new internet-delivered CBT module (from e-couch; www. ecouch.anu.edu.au) compared to an online CBT intervention (MoodGYM), "

Hypotheses:
"We hypothesized that the internet-delivered module of IPT would be noninferior to a CBT module in reducing symptoms of depression and anxiety. We also predicted that the Internet-delivered module of IPT would be rated as being as feasible, acceptable and satisfactory as the CBT module, and that the IPT and CBT modules would produce comparable effects to the MoodGYM intervention. "

**3b) CONSORT: Important changes to methods after trial commencement (such as eligibility criteria), with reasons**

NO

**3b-i) Bug fixes, Downtimes, Content Changes**

No bug fixes etc.

**4a) CONSORT: Eligibility criteria for participants**

"Individuals who were 18 years of age or older and not currently receiving treatment for depression by a mental health specialist were eligible for inclusion in the study. Individuals with suicide intention as measured with a suicidal ideation screening item on the Web Screening Questionnaire (WSQ [27]) or those who scored above 27 (95th percentile or higher) on the CES-D at baseline, were immediately provided with an information page containing advice about obtaining appropriate professional help, including emergency help. They could, however, continue to participate in the study. Excluded were individuals who were health professionals treating people with depression or anxiety, researchers reviewing depression or anxiety sites, or students studying anxiety or depression as part of a college or university course."

**4a-i) Computer / Internet literacy**

we did not explicitly asked about computer/internet literacy.

**4a-ii) Open vs. closed, web-based vs. face-to-face assessments:**

recruitment was online. everything was completely online.

"This automated, three-arm, fully self-guided online noninferiority trial compared two new treatments (IPT and CBT) to an active control treatment (MoodGYM) for depressed individuals. There was no specific promotion for the trial. Spontaneous visitors from around the world who registered on the Internet website e-couch (www.ecouch.anu.edu.au ) between October 2009 and October 2010 and who showed interest in participating (by clicking the 'I want more information about the trial' button) in the research trial, were given information about the study. Those who provided both informed consent to participating in the trial (by clicking on the 'I agree' button on the webpage) and an email address were then asked to complete an online baseline screening survey. "

"Individuals were not required to provide their real names, but instead were asked to use a pseudonym. "

**4a-iii) Information giving during recruitment**

yes see previous item.

**4b) CONSORT: Settings and locations where the data were collected**

"Spontaneous visitors from around the world who registered on the Internet website e-couch (www.ecouch.anu.edu.au ) between October 2009 and October 2010 "

**4b-i) Report if outcomes were (self-)assessed through online questionnaires**

online and selfassessed:

"were then asked to complete an online baseline screening survey."

**4b-ii) Report how institutional affiliations are displayed**

this was not mentioned in ms.

**5) CONSORT: Describe the interventions for each group with sufficient details to allow replication, including how and when they were actually administered**

**5-i) Mention names, credential, affiliations of the developers, sponsors, and owners**

"The interventions investigated in the current study were developed at the Centre for Mental Health Research, The Australian National University. H.C. and K.G. are authors and developers of the MoodGYM and eCouch websites but derive no personal or financial benefit from their operation. "

**5-ii) Describe the history/development process**

"(MoodGYM), which was originally developed for youth, but has known efficacy in reducing depressive symptoms in adults [22,23,24] Christensen et al., 2004; Farrer et al., 2011; Mackinnon et al, 2008). "

the new CBT and IPT programs were not evaluated before.

**5-iii) Revisions and updating**

"The standard online CBT package comprised a 4-module version of MoodGYM (moodgym.anu.edu.au) delivered over 4 weeks. The details of the program are described elsewhere [28,29]. The relaxation module was removed from the program for this study to equate the time length of the programs. Previous research has demonstrated that this component is not needed for efficacy [29]."

**5-iv) Quality assurance methods**

**5-v) Ensure replicability by publishing the source code, and/or providing screenshots/screen-capture video, and/or providing flowcharts of the algorithms used**

flow chart is provided, and URL to the ecouch website.

**5-vi) Digital preservation**

"The present study examined the effectiveness of internet-delivered IPT and a new internet-delivered CBT module (from e-couch; www.ecouch.anu. edu.au) "

**5-vii) Access**

no payment, no member of spec. group.

"There was no specific promotion for the trial. Spontaneous visitors from around the world who registered on the Internet website e-couch (www. ecouch.anu.edu.au ) between October 2009 and October 2010 and who showed interest in participating (by clicking the 'I want more information about the trial' button) in the research trial, were given information about the study. Those who provided both informed consent to participating in the trial (by clicking on the 'I agree' button on the webpage) "

"Following randomisation, an automated email containing log-in details for the assigned program was sent to each participant, at which point the intervention could be accessed immediately.'

**5-viii) Mode of delivery, features/functionalities/components of the intervention and comparator, and the theoretical framework**
"Internet-delivered Cognitive Behavioural Therapy (CBT)

The Internet-delivered CBT intervention comprised one ‘tool’ of the depression stream of e-couch (ecouch.anu.edu.au), an online program developed by the Centre for Mental Health Research (CMHR), The ANU. This intervention is based on the principles of CBT [12]. In addition to an explanation of the rationale of CBT, the program consists of three major modules, namely, identifying negative thoughts, tackling negative thoughts and undertaking behavioural activation, each of which contains eight exercises and assessments which are saved in a personal workbook. The program was offered over 4 weeks. Users were required to complete the CBT modules in order. Participants were able to revisit previous pages of the modules and scores of previous assessments, but were not able to repeat the assessments. Each week an automated email was sent to advise participants of the availability of their new module.

Internet-delivered Interpersonal Psychotherapy (IPT)

The internet-delivered form of IPT, comprised one ‘tool’ of the depression stream of e-couch (ecouch.anu.edu.au) which consists of four modules (grief, role disputes, role transition and interpersonal deficits). It also has a personal workbook (containing 11 exercises and assessments). The material is delivered over 4 weeks. Users were required to complete the modules in order. Participants were able to revisit previous pages of the modules and scores of previous assessments, but were not able to repeat the assessments. Each week an automated email was sent to advise participants of the availability of their new module.

Internet-delivered Standard CBT (MoodGYM)

The standard online CBT package comprised a 4-module version of MoodGYM (moodgym.anu.edu.au) delivered over 4 weeks. The details of the program are described elsewhere [28,29]. In this trial, a set of four of the CBT modules, a personal workbook (containing 22 exercises and assessments) and a feedback evaluation form were used. The modules cover the identification of and behavioral methods to overcome dysfunctional thinking, assertiveness and self-esteem training. Modules were undertaken sequentially. Each module undertakes 20-40 minutes [29]. Participants were able to revisit previous pages of the modules and scores of previous assessments, but were not able to repeat the assessments. Each week an automated email was sent to advise participants of the availability of their new module. The relaxation module was removed from the program for this study to equate the time length of the programs. Previous research has demonstrated that this component is not needed for efficacy [29].
"

**5-ix) Describe use parameters**
"The material is delivered over 4 weeks. Users were required to complete the modules in order. Participants were able to revisit previous pages of the modules and scores of previous assessments, but were not able to repeat the assessments. "

**5-x) Clarify the level of human involvement**
no level of human involvement. see previous answers.

**5-xi) Report any prompts/reminders used**
"Each week an automated email was sent to advise participants of the availability of their new module."

**5-xii) Describe any co-interventions (incl. training/support)**
no training/ support given

**6a) CONSORT: Completely defined pre-specified primary and secondary outcome measures, including how and when they were assessed**
"All questionnaires comprised standard self-report measures and were administered online. Measures were taken at baseline (pre-test), immediately after the intervention (post-test) and 6 months after the intervention (follow-up). Measures of participant characteristics were collected at baseline, symptoms measures were administered at all time points and user satisfaction was collected at post-test."

"Primary outcome measures: depressive symptoms

CES-D
The 20-item self-report Center for Epidemiological Studies Depression scale (CES-D; [30]) was used to assess depressive symptoms (item score: 0–3; total score range: 0–60). The Internet CES-D is reliable and valid with a cut-off score of 22 (Cronbach’s α: 0.92; AUC: 0.84; sensitivity: 0.94; specificity: 0.62 [31]). The Cronbach’s α in this study was 0.90. Since the CES-D was administered online, a cut-off score of 22 is used in this study.

Secondary outcomes: satisfaction & user perceived benefits and adherence:

CSQ-8

The Client Satisfaction Questionnaire (CSQ-8; [32]), assesses global client satisfaction with the treatment s/he is participating in. The 8-item self report questionnaire uses scale response options from 1 to 4, total score ranges from 8 to 32. Previous research has reported that the CSQ has high internal consistency (Cronbach’s α =0.93) and was comparable to the Cronbach’s α in this study (Cronbach’s α =0.90).

Adherence

Adherence was measured in two ways for each individual: (i) completion of post-test surveys (all groups); (ii) number of IPT, CBT or MoodGYM modules completed. "

**6a-i) Online questionnaires: describe if they were validated for online use and apply CHERRIES items to describe how the questionnaires were designed/deployed**
"The Internet CES-D is reliable and valid with a cut-off score of 22 (Cronbach’s α: 0.92; AUC: 0.84; sensitivity: 0.94; specificity: 0.62 [31]). The Cronbach’s α in this study was 0.90. "

**6a-ii) Describe whether and how “use” (including intensity of use/dosage) was defined/measured/monitored**
Logins/logfile were not reported, only adherence:

"In total, 30% completed post-test assessment and 28% completed follow-up assessment, while 25.8% and 16% was adherent to the program (completed 50% or more of the modules) and also completed post-test or follow-up assessment respectively.
Of the participants randomly assigned to IPT, 307 (49.5%) completed at least half of the intervention (two or more modules) and 169 (27.3%) completed all modules. For CBT, 230 subjects (37.7%) finished two or more modules and 88 (14.4%) completed all modules. A total of 195 participants (31.8%) finished half or more of the MoodGYM program. Of these, 67 subjects (10.9%) finished the whole program. "

**6a-iii) Describe whether, how, and when qualitative feedback from participants was obtained**

no qualitative feedback

**6b) CONSORT: Any changes to trial outcomes after the trial commenced, with reasons**

the Goldberg depr and anxiety scale was ommited from analyses (was a primary outcome measure) because it was not offered to the IPT/CBT participants (this was forgotten to be incorporated).

**7a) CONSORT: How sample size was determined**

**7a-i) Describe whether and how expected attrition was taken into account when calculating the sample size**

we recruited participants until we had enough people at post-test.

"Using 1-β=0.90 and α=0.05 (two-sided), we needed at least 150 participants in each condition at post-test (a total sample of 450 participants) to reach sufficient statistical power. "

**7b) CONSORT: When applicable, explanation of any interim analyses and stopping guidelines**

n.a.

**8a) CONSORT: Method used to generate the random allocation sequence**

"The randomisation schedule for participant allocation to condition groups was prepared using an automated system built into the trial software, and randomization occurred automatically"

**8b) CONSORT: Type of randomisation; details of any restriction (such as blocking and block size)**

"Participants were randomly assigned to MoodGYM, CBT or IPT, stratified by sex, age, and presenting depression symptom severity"

**9) CONSORT: Mechanism used to implement the random allocation sequence (such as sequentially numbered containers), describing any steps taken to conceal the sequence until interventions were assigned**

"The allocation sequence was concealed from the researchers. ."

**10) CONSORT: Who generated the random allocation sequence, who enrolled participants, and who assigned participants to interventions**

fully automatic: "automated system built into the trial software, and randomization occurred automatically"

**11a) CONSORT: Blinding - If done, who was blinded after assignment to interventions (for example, participants, care providers, those assessing outcomes) and how**

**11a-i) Specify who was blinded, and who wasn't**

"The allocation sequence was concealed from the researchers. Participants randomized to the intervention groups were aware of the allocated arm"

**11a-ii) Discuss e.g., whether participants knew which intervention was the "intervention of interest" and which one was the "comparator"**

there were 3 active treatments

**11b) CONSORT: If relevant, description of the similarity of interventions**

nr

**12a) CONSORT: Statistical methods used to compare groups for primary and secondary outcomes**

N/a/

**12a-i) Imputation techniques to deal with attrition / missing values**

intention to treat analyses using LMM.

"To conduct the per-protocol analysis, two groups were created: those who returned the post-test and follow-up surveys (completers), and those who completed half or more of the treatment modules and returned the surveys (adherent completers). The linear mixed models were used for both types of analyses. Restricted maximum likelihood estimation was used with an unstructured covariance structure accommodation with participant effects. LMM gives unbiased estimates of ITT effect under the assumption that data from participants who withdrew were missing at random (MAR). "

**12b) CONSORT: Methods for additional analyses, such as subgroup analyses and adjusted analyses**

"In addition, analyses were also undertaken for the sub-sample of participants who had symptoms severe enough to be considered clinical cases at baseline (those scoring ≥ 22 on the CES-D). "

"Results are presented for three groups: all participants (all those enrolled in the trial, intention-to-treat), completers (those completing online surveys at post-test, and 6 month follow-up), and adherent completers (ie, completers adherent to 50% of the modules)."

## RESULTS

**13a) CONSORT: For each group, the numbers of participants who were randomly assigned, received intended treatment, and were analysed for the primary outcome**

In the flowchart, the numbers of participants are shown who were assigned/received treatment and were analyzed for prim. outcome.

"Accordingly, a total of 1,929 participants were randomized to one of the three conditions. However, 66 of these participants were excluded after randomization because it became later apparent they were ineligible at baseline for participation (e.g., being a researcher or a student, n=21). In addition, 45 randomized participants did not complete the baseline assessment. This was missed at first screening due but picked up subsequently. Figure 1 shows the flow of participants through the trial. "

**13b) CONSORT: For each group, losses and exclusions after randomisation, together with reasons**

"In total, 30% completed post-test assessment and 28% completed follow-up assessment, while 25.8% and 16% was adherent to the program (completed 50% or more of the modules) and also completed post-test or follow-up assessment respectively.
Of the participants randomly assigned to IPT, 307 (49.5%) completed at least half of the intervention (two or more modules) and 169 (27.3%) completed all modules. For CBT, 230 subjects (37.7%) finished two or more modules and 88 (14.4%) completed all modules. A total of 195 participants (31.8%) finished half or more of the MoodGYM program. Of these, 67 subjects (10.9%) finished the whole program. Reasons given for drop-out included technical problems, personal issues (lack of time) disease-specific barriers (feeling too depressed to work on the program, not convinced that the program would help), general intervention problems (programs was taking too long, too much text to read, boring, too repetitive), specific intervention issues (the examples were not relevant to the subject), or engagement issues (preferred to obtain help from somewhere other than a computer). However, the majority of subjects did not provide any reason for drop-out. "

**13b-i) Attrition diagram**

no it does not include an attrition diagram, but this could be incorporated in a revised version. (The paper has already been submitted to JMIR).

**14a) CONSORT: Dates defining the periods of recruitment and follow-up**

"online surveys at post-test, and 6 month follow-up"

period of recruitment: " between October 2009 and October 2010"

**14a-i) Indicate if critical "secular events" fell into the study period**

no changes. / this is not mentioned in the MS.

**14b) CONSORT: Why the trial ended or was stopped (early)**

we had enough participants. this has been explained in the method section

**15) CONSORT: A table showing baseline demographic and clinical characteristics for each group**
table 1 with this relevant information is included.
**15-i) Report demographics associated with digital divide issues**
not known
**16a) CONSORT: For each group, number of participants (denominator) included in each analysis and whether the analysis was by original assigned groups**
**16-i) Report multiple "denominators" and provide definitions**
yes: "The intention-to-treat analyses yielded medium within-group effect sizes (d=0.59 to d=0.67 at post-test and d=0.66 to d=0.80 at follow-up). Between-group effect sizes were small (post-test: IPT vs. MoodGYM: d=0.09 (95% CI:-.02-0.21); CBT vs. MoodGYM: d=0.01 (95% CI: -.010-0.12); follow-up: IPT vs. MoodGYM: d=0.09 (95% CI: -.02-0.21), CBT vs. MoodGYM: d= 0.03 [95% CI: -0.08-0.14]). For completers, the within-group effect sizes on the primary outcome measure CES-D were large for all treatments at post-test (d=0.76 (IPT) to d=0.87 [CBT]) and follow-up (d=1.02 (IPT) to d=1.44 [CBT]). Between-group effect sizes were small (post-test: IPT vs. MoodGYM: d=0.14 (95% CI:-0.06-0.35); CBT vs. MoodGYM: d=0.05 (95% CI: -0.17-0.26); follow-up: IPT vs. MoodGYM: d= 0.18 (95% CI:-0.09-0.45), CBT vs. MoodGYM: d=0.12 [95% CI-0.15-0.39]). Within-group effect sizes for adherent completers ranged from d=0.74 to d=0.90 at post-test and d=1.02 to d=1.33 at follow-up. The between-group effect size for IPT vs. MoodGYM was higher (post-test: d=0.23 (95% CI: 0.0-0.46); follow-up: d=0.31[95% CI: 0.02-0.60]) than that for CBT vs. MoodGYM (post-test: d=0.02 (95% CI: -0.25-0.22); follow-up: d=0.04 [95% CI:-0.26-0.34]). See Table 2 and See Multimedia Appendix 1."
**16-ii) Primary analysis should be intent-to-treat**
"The intention-to-treat analyses yielded medium within-group effect sizes (d=0.59 to d=0.67 at post-test and d=0.66 to d=0.80 at follow-up). "
**17a) CONSORT: For each primary and secondary outcome, results for each group, and the estimated effect size and its precision (such as 95% confidence interval)**
"Between-group effect sizes were small (post-test: IPT vs. MoodGYM: d=0.09 (95% CI:-.02-0.21); CBT vs. MoodGYM: d=0.01 (95% CI: -.010-0.12); follow-up: IPT vs. MoodGYM: d=0.09 (95% CI: -.02-0.21), CBT vs. MoodGYM: d= 0.03 [95% CI: -0.08-0.14]). "
**17a-i) Presentation of process outcomes such as metrics of use and intensity of use**
"Results are presented for three groups: all participants (all those enrolled in the trial, intention-to-treat), completers (those completing online surveys at post-test, and 6 month follow-up), and adherent completers (ie, completers adherent to 50% of the modules)."
**17b) CONSORT: For binary outcomes, presentation of both absolute and relative effect sizes is recommended**
na
**18) CONSORT: Results of any other analyses performed, including subgroup analyses and adjusted analyses, distinguishing pre-specified from exploratory**
is provided, see previous answers.
**18-i) Subgroup analysis of comparing only users**
na
**19) CONSORT: All important harms or unintended effects in each group**
unknown.
**19-i) Include privacy breaches, technical problems**
N.a.
**19-ii) Include qualitative feedback from participants or observations from staff/researchers**
na

## DISCUSSION

**20) CONSORT: Trial limitations, addressing sources of potential bias, imprecision, multiplicity of analyses**
**20-i) Typical limitations in ehealth trials**
"Limitations
This study has several limitations. First, as mentioned above, the effect size found in this study differs from that on which we based the noninferiority margin and power calculation. To be able to draw reliable conclusions of noninferiority, it is important to establish effect sizes of similar size to prior trials. To minimize bias, it is important to replicate the conditions under which the control treatment was previously examined (e.g., the same population sample, outcome measures, assessment time points, analysis and delivery of treatment). MoodGYM is automated and has fidelity as an intervention. We have no reason to assume that omission of the MoodGYM relaxation module accounts for the difference in the effect size found in this study, as removal did not affect treatment effectiveness in a previous dismantling study [29]. The most likely cause of the observed differences lies in the different samples recruited. This aside, we can conclude that internet-delivered IPT is likely to be an effective treatment for depressive symptoms, and thereby offers people with depression another online treatment option. Second, the noninferiority margin of the primary outcome measure is usually based on the lower bound CI of the between group effect size of the traditional treatment [33]. In our case, this would be an effect size of 0.33 and a lower bound 95% CI of 0.11 [24]. To reach sufficient statistical power to be able to detect a significant difference, we would need at least 14,000 participants per condition. Therefore, we used an alternative approach to calculate the lower bound noninferiority margin. Based on the study of Mackinnon et al. [24]) we used the within-group effect size of 0.56 instead. This resulted in a noninferiority margin of an effect size of 0.33, which is a 3.795 difference on the CES-D. Although this difference is still liberal, an effect size of 0.30 is considered as the minimum for clinical meaningful change [49]. Third, there was a high drop-out rate. One possible explanation of the difference in attrition rates across the programs might be that MoodGYM takes longer to complete compared to the other programs, and lengthier programs might be associated with greater attrition [29]. Although MoodGYM had the highest drop-out rate, drop-out rates are, high amongst all conditions, a finding that is common for Internet interventions. Higher attrition and drop-out rates are likely with minimal exclusion criteria, unguided interventions [41,42], and little or no financial commitment [48]. However, a recent study of Hilvert-Bruce [50] showed that non-completers derive benefit before dropping out. Adding reminders, choice of course and timing, and financial cost can significantly improve adherence, and clinician contact during the course is also associated with increased adherence [50]. There were significant baseline differences (CES-D score, gender, age) between participants who completed the programs and those who did not, which might indicate selection bias. Intention-to-treat analyses nevertheless demonstrated effects. Finally, in face-to-face IPT, one foci is chosen, whereas in the internet-delivered IPT, all modules are undertaken by the participant. "

**21) CONSORT: Generalisability (external validity, applicability) of the trial findings**
**21-i) Generalizability to other populations**
our recruitment method was set up with limited exclusion criteria, no advertisement and used spontanous visitors (from the general population) of the ecouch website. this will be added to the revised paper.
**21-ii) Discuss if there were elements in the RCT that would be different in a routine application setting**
this will be added to the revised paper.
**22) CONSORT: Interpretation consistent with results, balancing benefits and harms, and considering other relevant evidence**
**22-i) Restate study questions and summarize the answers suggested by the data, starting with primary outcomes and process outcomes (use)**

"The present study is as far as we know the first to show that internet-delivered IPT can be as effective as internet-delivered CBT in the treatment of depressive symptoms at post-test and 6-month follow-up. Both the IPT and the CBT online interventions employed in the trial showed medium to large and significant within-group effect sizes on the CES-D for completers and adherent completers. For the intention-to-treat sample, effect-sizes were smaller but still moderate to large in size. Of the clinical cases, completers and adherent completers showed medium to large effect sizes on post-test and follow-up ratings. We found that IPT and CBT were noninferior compared to MoodGYM for those who returned post-test, and between group effect sizes were small"

**22-ii) Highlight unanswered new questions, suggest future research**

"Future research is needed to replicate noninferiority compared to CBT programs and to test whether the new CBT program is superior to other programs, to examine whether guided internet-delivered IPT is as effective as face-to-face IPT, to investigate methods to improve adherence, and whether internet-delivered IPT is also effective in the treatment for other disorders, such as social phobia or panic disorder. It is important that future research investigates individual characteristics, such as recent life events, that predict treatment response for IPT. There will also be value in investigating whether a planned extended version of e-couch IPT will yield higher satisfaction ratings."

## Other information

**23) CONSORT: Registration number and name of trial registry**

"Trial Registration: ISRCTN69603913"

**24) CONSORT: Where the full trial protocol can be accessed, if available**

Not available

**25) CONSORT: Sources of funding and other support (such as supply of drugs), role of funders**

"This study was funded by the Faculty of Psychology and Education of the VU University, Amsterdam and the Centre for Mental Health Research at The Australian National University. HC is supported by NHMRC Fellowship 525411. KG is supported by an NHMRC Senior Research Fellowship No. 525413. "

**X26-i) Comment on ethics committee approval**

"Ethical approval for the study was provided by the Human Ethics Committee of the Australian National University (ANU). "

**x26-ii) Outline informed consent procedures**

"Those who provided both informed consent to participating in the trial (by clicking on the ‘I agree’ button on the webpage) "

**X26-iii) Safety and security procedures**

safety: "Individuals with suicide intention as measured with a suicidal ideation screening item on the Web Screening Questionnaire (WSQ [27]) or those who scored above 27 (95th percentile or higher) on the CES-D at baseline, were immediately provided with an information page containing advice about obtaining appropriate professional help, including emergency help. They could, however, continue to participate in the study. "

privacy: "Individuals were not required to provide their real names, but instead were asked to use a pseudonym. "

**X27-i) State the relation of the study team towards the system being evaluated**

"The interventions investigated in the current study were developed at the Centre for Mental Health Research, The Australian National University. H.C. and K.G. are authors and developers of the MoodGYM and eCouch websites but derive no personal or financial benefit from their operation. "
