# Supplementary figures and images for: Internet-Delivered Interpersonal Psychotherapy Versus Internet-Delivered Cognitive Behavioral Therapy for Adults With Depressive Symptoms: Randomized Controlled Noninferiority Trial
Source: J Med Internet Res. 2013 May 13;15(5):e82. doi: 10.2196/jmir.2307 (PMC3668608; doi:10.2196/jmir.2307)

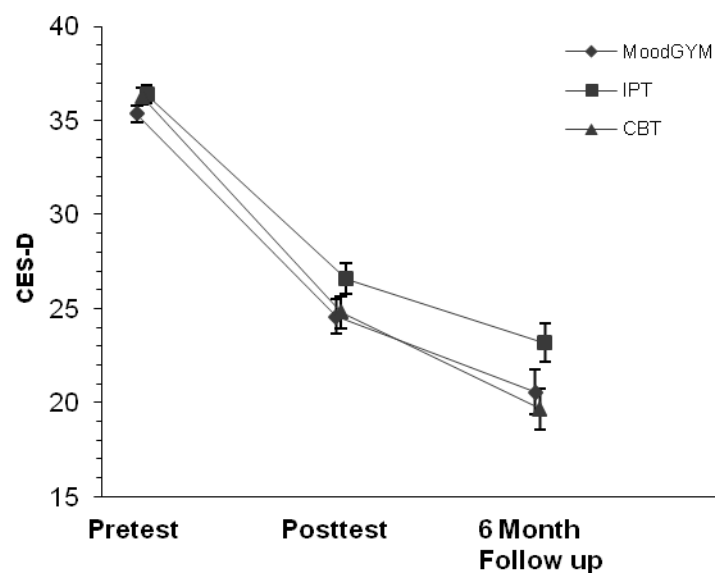

Supplement: Supplementary file 2 [file jmir_v15i5e82_app2.pdf]
