# Supplementary material for: Internet-Delivered Interpersonal Psychotherapy Versus Internet-Delivered Cognitive Behavioral Therapy for Adults With Depressive Symptoms: Randomized Controlled Noninferiority Trial
Source: J Med Internet Res. 2013 May 13;15(5):e82. doi: 10.2196/jmir.2307 (PMC3668608; doi:10.2196/jmir.2307)
Supplement: Supplementary file 3 [file jmir_v15i5e82_app3.pdf]

| **Program** | **Test time, n;mean (SD)** | | | **Within-group effect size *d* (95% CI)** | | **Between-group effect size *d* (95% CI)** | | |
|---|---|---|---|---|---|---|---|---|
| | Pretest | Posttest | Follow-up | Pre- Post | Pre-Follow-up [a] | Program | Posttest | Follow-up |
| **Completers of posttest (n=478) and/or follow-up (n=245)** | | | | | | | | |
| IPT | 181; 38.57 (9.20) | 181; 27.89 (12.42) | 93; 24.04 (14.39) | 0.98 (0.76, 1.19) | 1.20 (0.88, 1.50) | IPT vs Mood-GYM | 0.09 (-0.14,0.31)[b] | 0.17 (0.15, 0.49) [b] |
| CBT | 160; 37.12 (9.00) | 160; 25.51 (12.93) | 88; 19.50 (12.59) | 1.04 (0.81, 1.27) | 1.60 (1.25, 1.93) | CBT vs Mood-GYM | 0.10 (-0.32, 0.13)[b] | 0.14 (-0.18,0.47) [c] |
| MoodGYM | 137 39.06 (8.93) | 137; 26.78 (13.53) | 64; 21.50 (15.45) | 1.07 (0.82, 1.32) | 1.36 (0.97, 1.74) | | | |
| **Adherent completers of posttest (n=412) and/or follow-up (n=252)** | | | | | | | | |
| IPT | 168; 38.60 (8.96) | 168; 28.13 (12.72) | 102; 23.97 (13.78) | 0.95 (0.72,1.17) | 1.25 (0.95,1.55) | IPT vs Mood-GYM | 0.14 (-0.11-0.38)[b] | 0.24 (-0.08, 0.55) [b] |
| CBT | 140; 36.83 (8.96) | 140; 24.76 (12.71) | 89; 18.97 (13.78) | 1.10 (0.84,1.35) | 1.54 (1.20,1.87) | CBT vs Mood-GYM | 0.09 (-0.16-0.35)[c] | 0.13 (-0.20, 0.45) [c] |
| MoodGYM | 104; 38.76 (8.95) | 104; 26.11 (17.15) | 61; 20.71 (13.77) | 0.92 (0.64,1.21) | 1.49 (1.08,1.89) | | | |
| **ITT posttest and follow-up (N=1624** | | | | | | | | |
| IPT | 542; 38.57 (15.67) | 542; 27.89 (22.35) | 542; 24.54 (30.57) | 0.55 (0.43, 0.67) | 0.58 (0.46,0.70) | *IPT vs* Mood-GYM | 0.05 (-0.07, 0.17)[b] | 0.08 (-0.04, 0.20) [b] |
| CBT | 555; 37.12 (16.87) | 555; 25.51 (24.05) | 555; 20.35 (32.06) | 0.56 (0.44, 0.68) | 0.65 (0.53, 0.77) | *CBT vs* Mood-GYM | 0.05 (-0.07,0.17)[c] | 0.04 (-0.08, 0.16) [c] |
| MoodGYM | 527; 39.06 (17.75) | 527; 26.78 (25.32) | 527; 21.74 (35.90) | 0.56 (0.44, 0.68) | 0.61 (0.49, 0.73) | | | |

[a]Within-group follow-up effect size for completers is based upon the following pre-test scores: IPT (n=93, mean 38.72, SD 9.66); CBT: ( n=88, mean 37.25, SD: 9.30); MoodGYM (n=64, mean 38.58, SD: 8.68). Within-group follow-up effect size for adherent completers is based upon the following pre-test scores: IPT (n=102, mean 38.69, SD: 9.29); CBT ( n=89, mean 37.12 , SD: 9.30); MoodGYM (n=61, mean 38.26, SD: 9.29).

[b] in favor of MoodGYM

[c] in favor of CBT.
